# The Effects of *Berberis integerrima* Fruit Extract on Glycemic Control Parameters in Patients with Type 2 Diabetes Mellitus: A Randomized Controlled Clinical Trial

**DOI:** 10.1155/2021/5583691

**Published:** 2021-07-05

**Authors:** Rasool Soltani, Syed Mustafa Ghanadian, Bijan Iraj, Alireza Homayouni, Tanin Shahmiveh Esfahani, Mojtaba Akbari

**Affiliations:** ^1^Department of Clinical Pharmacy and Pharmacy Practice, Faculty of Pharmacy and Pharmaceutical Sciences, Isfahan University of Medical Sciences, Isfahan, Iran; ^2^Infectious Diseases and Tropical Medicine Research Center, Isfahan University of Medical Sciences, Isfahan, Iran; ^3^Isfahan Pharmaceutical Research Center, Faculty of Pharmacy and Pharmaceutical Sciences, Isfahan University of Medical Sciences, Isfahan, Iran; ^4^Isfahan Endocrine and Metabolism Research Center, Isfahan University of Medical Sciences, Isfahan, Iran; ^5^Department of Pharmaceutics, Faculty of Pharmacy and Pharmaceutical Sciences, Isfahan University of Medical Sciences, Isfahan, Iran; ^6^Research and Development Department, Goldaru Pharmaceutical Company, Isfahan, Iran; ^7^Students Research Committee, Faculty of Pharmacy and Pharmaceutical Sciences, Isfahan University of Medical Sciences, Isfahan, Iran; ^8^Department of Epidemiology and Statistics, Faculty of Medicine, Isfahan University of Medical Sciences, Isfahan, Iran

## Abstract

**Background:**

*Berberis integerrima* Bunge fruits have been utilized in traditional medicine to control diabetes mellitus (DM). However, no clinical survey has been done in this regard. This study was conducted to clinically evaluate the effects of fruit extract of this plant in improving glycemic control indices in patients with type 2 DM (T2DM).

**Methods:**

In a randomized controlled clinical trial, patients with T2DM who met the inclusion criteria were randomly divided into two groups of drug (*Berberis*) and control to receive the extract solution 5 ml twice daily (equivalent to 1000 mg of dry extract) with standard treatment (metformin) or only standard treatment, respectively, for 8 weeks. Before and after the intervention, fasting blood sugar (FBS), serum glycosylated hemoglobin (HbA1c), serum insulin, the homeostasis assessment model for insulin resistance (HOMA-IR), body mass index (BMI), and systolic and diastolic blood pressure were determined and compared between the two groups.

**Results:**

During the study, 30 and 35 patients in the drug and control groups, respectively, completed the study. Although no significant changes occurred in any parameter within each group, postintervention FBS (117.5 [107–128.8] versus 134 [120–142], *P* = 0.001) and HbA1c (7 [6.4–7.7] versus 7.5 [6.8–7.9], *P* = 0.045) were significantly lower in the drug group than in the control one. In terms of other parameters, there were no significant differences between the groups.

**Conclusion:**

Consumption of *B. integerrima* Bunge fruit extract at a dosage of 1000 mg daily decreases FBS and HbA1c but does not affect HOMA-IR in patients with type 2 diabetes mellitus.

## 1. Introduction

Diabetes mellitus (DM), including type 2 DM (T2DM), is one of the most common endocrine disorders that, if left untreated, can lead to neurological, cardiovascular, and renal complications as well as foot ulcers and subsequent infection [1].

Proper treatment for glycemic control is imperative to slow the devastating complications of DM [2]. Drugs used to treat T2DM have several adverse effects including hypoglycemia, obesity, allergic reactions, gastrointestinal disorders (anorexia, nausea, and vomiting), lactic acidosis, and anemia [3]. On the other hand, many patients remain hyperglycemic despite pharmacotherapy with combination of drugs [4]; treatment-resistant T2DM is now a common challenge in clinical practice. Therefore, finding new drugs or supplements for adjunctive therapy is mandatory to reduce the required dose of antidiabetic drugs and consequent adverse effects and to improve glycemic control in difficult-to-treat cases.

The use of herbal medicines has increased due to the public idea of fewer side effects and lower cost [5]. So far, the positive effects of many herbs in lowering blood glucose or reducing the side effects of hyperglycemia have been known [6]. The antidiabetic effects of plants are attributed to their phytochemicals including phenolic compounds, flavonoids, terpenoids, and alkaloids that have shown hypoglycemic effects [7].

*Berberis* plants (commonly known as barberry), including *Berberis integerrima* Bunge, from the Berberidaceae family [8] are used in the traditional medicine of some countries, including Iran, China, Pakistan, and India, to control blood sugar [9]. Barberry fruit has antioxidant [10], anti-inflammatory [11], antihypertensive [12], hypoglycemic [13], and lipid-lowering [14] effects. *B. integerrima* fruits are rich in compounds such as berberine, berbamine, protoberberine, polyphenols, anthocyanins, and pectin [15]. Berberine has been suggested to lower blood glucose by several mechanisms including the increase in insulin sensitivity, stimulating glycolysis in peripheral tissues, inhibiting gluconeogenesis in the liver, and inhibiting the gastrointestinal absorption of carbohydrates [16, 17]. Furthermore, the anthocyanins in barberry fruit may also have a role in its hypoglycemic effect [18].

Some animal studies have shown the antidiabetic effect of *B. integerrima* fruits [18, 19]. However, to the best of our knowledge, no clinical study has been done in this regard. Therefore, the present study was performed to clinically evaluate the effects of fruit extract of this plant in improving glycemic control indices in patients with type 2 diabetes.

## 2. Materials and Methods

### 2.1. Extraction

Fresh fruits of *B. integerrima* were purchased from the nearby market in the southern Khorasan province and confirmed by a taxonomist in Ferdowsi University of Mashhad with voucher number SAM-1890 deposited in Samsam-Shariat Herbarium, Pharmacognosy Department, Isfahan University of Medical Sciences (IUMS).

Plant material (15 kg) was cut into modest pieces twice by a crushing machine. Extraction was done with ethanol : water (6 : 4) in maceration tank for four days with three repetitions. The extract was then concentrated with a rotary evaporator (Heidolph, Germany) and stored in a refrigerator. To determine the percentage of the dry weight of the extract, a crucible was placed in an oven at 110°C for 20 minutes and weighed after cooling in a desiccator. Then, 1 g of the weighed plant extract was poured into the crucible and placed in the oven again. After one hour, the crucible was cooled in the desiccator and weighed (by three times). Using the following formulas, the extraction yield was calculated as 46.7% (*w*/*w*).

(1) $$\begin{array}{c} \text{Dry weight }\%=\left(\frac{\text{dry weight}}{\text{extract weight}}\right)\times 100;\\ \text{Extraction yield}=\left(\frac{\text{dry weight percentage}\times \text{total amount of concentrated extract}}{\text{fruit weight}}\right)\times 100. \end{array}$$

### 2.2. Extract Standardization by Total Phenolic and Total Flavonoid Content

For standardization of the extract, the content of polyphenols was determined by Folin-Ciocalteu method [20]. For this purpose, 1 g of the extract was transferred to a volumetric flask and reached the volume of 10 ml with methanol. Then, to 20 *μ*l of the sample, 1.58 ml of water, 100 *μ*l of the Folin-Ciocalteu reagent, and 300 *μ*l of 20% sodium carbonate solution were added and shaken well to be completely mixed. The solution was kept at 20°C for 2 hours in a dark environment, and then the absorption was read at 765 nm against blank. The gallic acid solution was used as the standard for polyphenolic compounds. After preparing different concentrations of gallic acid solution, absorption reading at 765 nm, and drawing the standard curve(*y* = 0.0551*x* + 0.0303), total phenol content (TPC) of the extract was calculated as 86.88 ± 0.246 mg gallic acid equivalent per gram of dry extract.

The total flavonoid content (TFC) of dry extract was calculated using spectrometry method of AlCl3-flavonoid complex as reported before [20]. Briefly, a 10% diluted solution of dry extract (100 *μ*L) was added to the test tubes containing 100 *μ*L of 20% AlCl3 and 20 *μ*L of glacial acetic acid, and methanol was added to 3 mL. After 30 min of incubation, the absorbance was read at 415 nm. Based on the quercetin calibration formula, *y* = 0.046*x* − 0.0086, TFC of the extract was calculated as 3.88 ± 0.0.17 mg quercetin equivalent per gram of dry extract.

### 2.3. Preparation of Oral Solution (Syrup) from the Extract

To prepare barberry syrup, a mixture of 8.5 g of concentrated extract with several solvents and excipients including water, 70% ethanol, polyethylene glycol (PEG), tween, pectin, and poloxamer was tested in different ratios to obtain a clear and stable solution. Finally, the clearest and the most stable solution with the following ingredients and ratios (*w*/*v*) was selected for administration to the patients: carboxymethylcellulose (CMC) 0.5%, aspartame 1.5%, poloxamer 2.5%, polyethylene glycol 12.5%, citric acid 2%, and distilled water 100 ml. Furthermore, the orange extract was used as the flavoring agent.

### 2.4. Study Design

This study was a randomized controlled clinical trial conducted from September 2018 to April 2020 in the Faculty of Pharmacy and Isfahan Endocrine & Metabolism Research Center (IEMRC), both affiliated to IUMS, Isfahan, Iran. The study was approved by the ethics committee of IUMS with the ethics code IR.MUI.RESEARCH.1398.104 and registered at Iranian Registry of Clinical Trials with the code IRCT20150721023282N5.

### 2.5. Patients and Interventions

Participants were selected from diabetic patients referred to the diabetes clinic of IEMRC. All patients were met prior to participation to become utterly familiar with the study, and, if agreed, their consent was obtained.

The study inclusion criteria were (1) age between 18 and 75 years; (2) being diagnosed with type 2 diabetes (according to the diagnostic criteria of the American Diabetes Association) for at least 2 years; (3) treatment with metformin; (4) glycosylated hemoglobin (HbA1c) between 6.5 and 9%; (5) not consuming alcohol and other abused substances; (6) no liver or kidney disease; (7) not taking oral antidiabetic drugs such as sulfonylureas and glinides and insulin products; (8) no pregnancy and lactation.

Patients were excluded from the study if they did not follow the medication instructions for more than three days, developed allergic reaction to barberry extract during the study, or changed their treatment for diabetes (change in dose, type, or the number of medications) during the study.

By convenience sampling, all subjects who met the inclusion criteria were consecutively chosen and randomly divided into two groups of drug (barberry extract) and control. Block randomization method was utilized for randomization. In this way, all possible blocks of 4 were numbered and after that, using random number table, the specified blocks were chosen and the individuals of each group were selected based on the arrangements within the blocks.

Demographic characteristics of patients including age, sex, height, weight, BMI, and systolic (SBP) and diastolic (DBP) blood pressure were recorded. Appropriate diet and maintenance of physical activity were suggested to both groups during the study. Dietary instructions were given by a qualified dietician. Before starting treatment, 10 ml of venous blood sample was taken from each patient in the two groups in the fasting state (8 to 12 hours overnight) centrifuged within a maximum of 30 to 45 minutes of sampling, and serum glucose (FBS), HbA1c, and insulin levels were determined (due to the experiments of all patients in one step, the centrifuged samples were stored at freezer −70°C until assays). Moreover, using the following formula, the homeostasis assessment model for insulin resistance (HOMA-IR) was calculated and recorded as an indicator of insulin resistance:

(2) $$\begin{array}{c} \text{HOMA}-\text{IR}=\frac{\left[\text{fasting insulin}\left(µ\text{U}/\text{mL}\right)\times \text{fasting glucose}\left(\text{mmol}/\text{L}\right)\right]}{22.5}. \end{array}$$

For patients of the drug group, in addition to the standard treatment according to the physician's opinion, barberry extract syrup was administered at a dose of 5 ml twice a day (equivalent to 1000 mg of prepared dry extract) for eight weeks. The control group received only standard treatment. At the end of the 8th week of treatment, by taking blood samples from the two groups, the mentioned indicators were measured and recorded again. Finally, the recorded results of the two groups were compared with appropriate statistical tests.

Patients were asked to report any side effects from the syrup. Furthermore, in order to evaluate the patients' compliance for interventions including diet and physical activity issues, they were contacted by telephone every other day and visited in the third week of intervention, when, for participants of drug group, the previous syrup bottle was taken and evaluated in terms of the amount of consumed content, and a new bottle was given to them.

### 2.6. Outcome Measures

The primary outcome measures were the changes in FBS, HbA1c, serum insulin levels, and HOMA-IR, while the secondary outcome measures included the changes in BMI, SBP, and DBP at the end of intervention compared to the baseline values.

### 2.7. Sample Size Calculation

Considering that the main variables of this study were continuous quantitative type, the following formula was used for sample size calculation:

(3) $$\begin{array}{c} n=\frac{{\left(Z_{\alpha /2}+Z_{\beta }\right)}^{2}\times 2{\text{SD}}^{2}}{d^{2}}, \end{array}$$

where *n* is the required sample size in each group, *d* is the clinically important difference between the groups, SD is the standard deviation, *Z*_*α*/2_ is the standard normal *z*-value for a significance level *α* = 0.05, which is 1.196, and *Z*_*β*_ is the standard normal *z*-value for the power of 80%, which is 0.84. According to HbA1c values in a previous study [21], the SD and *d* quantities were considered 0.6 and 0.5, respectively. Therefore, a calculated sample size of at least 22 patients in each group was obtained.

### 2.8. Statistical Analysis

SPSS software version 24 was used to perform statistical analysis. Quantitative data were reported as mean ± SD or median (IQR) and qualitative data as number and percent. Due to nonnormal distribution of data, Wilcoxon signed-rank test was used to compare the preintervention and postintervention values within each group, while the Mann–Whitney *U* test was applied for comparison of values in each time point between the groups. Analysis of covariance (ANCOVA) was used to compare the changes in the parameters between the two groups. The significance level was considered to be *P* < 0.05.

## 3. Results

### 3.1. Patients

**Figure 1:**
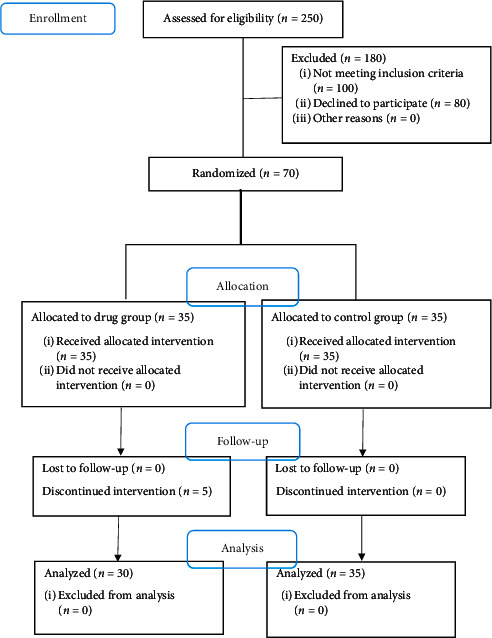
The CONSORT flowchart of the study.

During the study, out of 250 patients surveyed, 150 patients met the inclusion criteria, of whom 70 were willing to cooperate and were randomized to the groups. During the study, 5 people from the drug group were excluded from the study due to either side effects of the syrup after the first doses (2 patients) or reluctance to continue the participation (3 patients). Finally, 30 and 35 patients in drug and control groups, respectively, completed the study (Figure 1). The baseline characteristics of patients are shown in Table 1. As seen, there was no significant difference between the groups regarding age, sex distribution, and consumed antidiabetic drug (other than metformin).

### 3.2. Efficacy Evaluation

Table 2presents the changes of the evaluated parameters in each group along with the comparison of their values between the two groups. As can be seen, even though no significant change occurred in any parameter within each group, a statistically significant difference was observed between the two groups regarding postintervention FBS (*P* = 0.001) and HbA1c (*P* = 0.045); both parameters were significantly lower in the drug group than in control as confirmed by ANCOVA with the control of baseline values. In terms of other parameters, there were no significant differences between the groups.

### 3.3. Side Effects

During the study, only two patients in the drug group complained of heartburn and weakness (one patient in each case) who were excluded from the study. The other patients who completed the study did not report any side effects.

## 4. Discussion

In this study, in the group receiving barberry syrup, at the end of the eighth week, there was a significant decrease in FBS and HbA1c compared to the control group as well as a slight increase in fasting insulin and HOMA-IR which was not significant compared to the control group with a higher increase of these parameters. In addition, consumption of this extract did not affect BMI and blood pressure. According to our research, this is the first human study of *B. integerrima* fruits in diabetic patients, and other studies have been animal type with different results.

In an animal study performed on diabetic rats, the effects of *B. integerrima* fruit extract (1000 mg/kg) on blood sugar and serum insulin levels were investigated. Based on the results, at the end of the eighth week, weight gain, insulin and blood glucose levels, and HOMA-IR decreased significantly [18]. In a study on streptozotocin-induced diabetic rats, the anthocyanin fraction extracted from *B. integerrima* fruit reduced blood glucose levels and body weight compared with untreated diabetic rats [18]. Therefore, it seems that barberry fruit anthocyanins play a major role in its hypoglycemic effects.

Despite the lack of a clinical study on the effect of *B. integerrima* fruits on type 2 diabetes mellitus, in a recent survey by Sanjari et al., the use of *B. integerrima* root extract (480 mg/day) by T2DM patients resulted in a significant reduction of FBS, HbA1c, and 2-hour postprandial glucose (2-hPG) equivalent to metformin [22]. Consistent with our results, this study shows the beneficial effects of this species of *Berberis* on T2DM. In addition, in a recently published clinical study, the effects of *B. integerrima* fruit extract (at a dose of 1500 mg per day for 3 months) on FBS and several nonglycemic indices were evaluated in patients with rheumatoid arthritis treated with glucocorticoids. According to the results, the consumption of this extract was associated with a significant reduction in FBS [23]. This is in agreement with the results of our study. However, since the patients were not diabetic in the mentioned study, the mean FBS values were lower than ours. Therefore, it seems that the hypoglycemic effects of barberry could occur even in nondiabetic patients.

There are several studies on the hypoglycemic effects of *Berberis vulgaris*, another species of barberry. However, as the total phenolic content and antioxidant activity of *B. integerrima* are higher than those of *B. vulgaris* [24], the first plant could be a reasonable choice for this study. In the study of Shidfar et al., the effect of *B. vulgaris* fruit extract (at a dose of 3 g per day for 3 months) on the levels of insulin, blood glucose, and HbA1c in type 2 diabetic patients was investigated [25]. According to the results, consumption of the extract led to a significant reduction in FBS, which is consistent with our results. However, contrary to our results, there was no significant change in HbA1c levels, while insulin levels and HOMA-IR decreased significantly. Therefore, it seems that the blood glucose-lowering effect is decisive for barberry species. However, the ideal dose and dose-response relationship for this effect should be determined in further studies. On the other hand, the effect of barberry extract on insulin level and insulin resistance index (HOMA-IR) has been different between the studies. In our study, insulin levels increased slightly, which could indicate that part of the hypoglycemic effect was due to increased insulin secretion. Since, in the study of Shidfar et al., contrary to our research, insulin levels and, consequently, HOMA-IR decreased, a similar effect might be observed with *B. integerrima* extract if the dose and duration of intervention increased. Of note, our evaluated dose was one-third of the dose prescribed in the above-mentioned study, and the duration of our study was one month less than that. In fact, hyperinsulinemia in type 2 diabetic patients is a compensatory response to insulin resistance-induced hyperglycemia resolving overtime after proper glucose control. Therefore, the lack of effect on insulin resistance index (HOMA-IR) in our study may be due to an insufficient effect on hyperglycemia or short intervention duration or both.

The hypoglycemic effect of barberry could be due to various mechanisms. In an animal study on diabetic rats, *B. vulgaris* resulted in increased insulin secretion and pancreatic beta cells and subsequent blood sugar reduction [26]. Because oxidative degradation of the pancreas plays a role in the T2DM development, antioxidant compounds such as barberry fruit extract [10] may be effective in reducing this damage and improving glycemic control [27]. Phenolic compounds in barberry play an essential role in its antioxidative effects [28]. Notably, as mentioned previously, the amount of these compounds in *B. integerrima* fruit is more than that in *B. vulgaris* [24]. Moreover, increased insulin secretion, as also seen in our study, can be a reason for lowering blood glucose by barberry [26].

One of the substances in *B. integerrima* is the alkaloid berberine [15]. The antidiabetic effects of berberine have been demonstrated in numerous studies [16]. Increased insulin sensitivity, activation of the AMPK (adenosine monophosphate-activated protein kinase) pathway, induction of glucagon-like protein-1 secretion from the intestine, stimulation of glycolysis in peripheral tissues, inhibition of gluconeogenesis in the liver, increase of glucose transporters in the cells, decrease in the activity of *α*-glucosidase, and consequent inhibition of carbohydrates absorption are some of the suggested mechanisms for this effect [16, 17].

The anthocyanins in barberry fruit may also have a role in its antidiabetic effect. In the study of Sabahi et al., anthocyanins of *B. integerrima* fruit decreased glucose levels, increased liver glycogen, and resulted in weight loss in the treated rats [18]. In the clinical study of Yang et al. on prediabetic and early untreated diabetic patients, use of purified anthocyanins (320 mg/day) significantly reduced HbA1c (-0.14%) [29]. The antioxidant properties of anthocyanins protect pancreatic beta cells against oxidative stress. Anthocyanins also release insulin from the pancreas and increase AMPK phosphorylation and activation of this enzyme, leading to increased glucose transport to the muscle cells and decreased serum glucose levels [18].

Although in the present study *B. integerrima* did not have a significant effect on body weight, BMI, and blood pressure in diabetic patients, the weight loss and hypotensive effects of *Berberis* plants have been shown previously [30, 31]. Furthermore, barberry is used in the traditional medicine to treat hypertension. Berberine has been suggested to have antiobesity effect by inhibiting adipogenesis [32]. Since the impacts of barberry extract on these parameters were not primary objectives of this study, more controlled studies are needed to evaluate such effects.

The main limitations of our study were relatively low sample size, lack of placebo and blinding (due to technical problems), and no precise control for patients' diet and calorie intake. However, this is the first pilot clinical study showing the beneficial effects of *B. integerrima* fruit extract on glycemic control of T2DM patients. Of note, more clinical studies with larger sample size and longer duration are necessary to confirm these effects.

## 5. Conclusion

Consumption of *B. integerrima* Bunge fruit extract at a dosage of 1000 mg daily decreases FBS and HbA1c but does not affect HOMA-IR in patients with type 2 diabetes mellitus. Therefore, this extract can be considered as a dietary supplement in the treatment of type 2 diabetes mellitus.

## Figures and Tables

**Table 1:** Baseline characteristics of study patients.

Parameter	Drug group (*Berberis*) (*n* = 30)	Control group (*n* = 35)	
Age (years; mean ± SD)	56.10 ± 7.20	57.60 ± 7.70	0.416
Sex (n)			
Male	5	6	0.959
Female	25	29	
Antidiabetic drug (*n*)^*∗*^			
Acarbose	1	1	
Sitagliptin	8	8	0.837
Linagliptin	1	1	
Pioglitazone	7	5	

^*∗*^Antidiabetic drug other than metformin.

**Table 2:** Preintervention and postintervention values of parameters and their comparison between the two groups.

Parameter	Group	Time		*P* value^*∗∗*^	OR (95% CI)^*δ*^
		Baseline	End (week 8)		
BMI (kg/m^2^)	Control	29.5 ± 4.4	29.6 ± 4.3	0.180	1.375 (0.619–3.058)
	*Berberis*	4.4 ± 29.7	4.4 ± 29.7	0.970	
	*P*value^*∗*^	0.856	0.935	0.492^*∗∗∗*^	

FBS (mg/dl)	Control	128 (118–141)	134 (120–142)	0.110	0.979 (0.956–1.002)
	*Berberis*	125 (114.8–134.5)	117.5 (107–128.8)	0.110	
	*P*value^*∗*^	0.498	0.001	0.026^*∗∗∗*^	

Serum insulin (U/ml)	Control	4.8 (3.1–10.1)	7.5 (3.8–9.4)	0.296	0.975 (0.881–1.079)
	*Berberis*	5 (2.9–8.6)	6 (3.6–9.1)	0.698	
	*P*value^*∗*^	0.963	0.400	0.792^*∗∗∗*^	

HbA1c (g/dl)	Control	7.2 (6.7–8.4)	7.5 (6.8–7.9)	0.872	1.413 (0.860–2.321)
	*Berberis*	7.4 (6.8–7.7)	7 (6.4–7.7)	0.430	
	*P*value^*∗*^	0.879	0.045	0.045^*∗∗∗*^	

HOMA-IR	Control	26.8 (14.9–54.5)	43.5 (23.3–55.1)	0.368	1.003 (0.987–1.020)
	*Berberis*	28.8 (15.4–46.1)	30.7 (17.9–46.4)	0.910	
	*P*value^*∗*^	0.803	0.124	0.517^*∗∗∗*^	

SBP (mm Hg)	Control	13 (12–14)	13 (12–14)	0.936	0.884 (0.592–1.319)
	*Berberis*	12 (11–14)	12 (11–14)	0.315	
	*P*value^*∗*^	0.450	0.234	0.246^*∗∗∗*^	

DBP (mm Hg)	Control	8 (7–9)	8 (7-8)	0.415	1.308 (0.754–2.268)
	*Berberis*	8 (7-8)	8 (7–9)	0.565	
	*P*value^*∗*^	0.521	0.994	0.298^*∗∗∗*^	

The values are mean ± SD for BMI and median (IQR) for other parameters. BMI, body mass index; FBS, fasting blood sugar; HbA1c, hemoglobin A1c; HOMA-IR, homeostasis assessment model for insulin resistance; SBP, systolic blood pressure; DBP, diastolic blood pressure. ^*∗*^Comparison of preintervention and postintervention values between the two groups (Mann–Whitney *U* test). ^*∗∗*^Comparison of preintervention and postintervention values within each group (Wilcoxon signed-rank test). ^*∗∗∗*^Comparison between the two groups with control of baseline values (ANCOVA test). ^*δ*^Odds ratio and 95% confidence interval for comparison of mean differences between the groups.

## Data Availability

Ethically, the obtained data are confidential as this work is a clinical trial. However, in necessary situations, the data will be sent to the reader via e-mail upon request.

## References

[1] Barrnett P. H., Braunstein G. D., Carpenter C., Grigges R. C., Loscatzo J. (2001). Diabets mellitus. *Cecil Essentials of Medicine*.

[2] Goldman J. D., Patel D. K., Schnee D., Zeind C. S., Carvalho M. G. (2018). Diabetes mellitus. *Applied Therapeutics: The Clinical Use of Drugs*.

[3] Chaudhury A., Duvoor C., Dendi V. S. R. (2017). Clinical review of antidiabetic drugs: implications for type 2 diabetes mellitus management. *Frontiers in Endocrinology*.

[4] Scheen A. J. (2017). Pharmacotherapy of ‘treatment resistant’ type 2 diabetes. *Expert Opinion on Pharmacotherapy*.

[5] Craig W. J. (1999). Health-promoting properties of common herbs. *The American Journal of Clinical Nutrition*.

[6] Ilahi I., Khan A., Ullah U., Khan I., Ali J., Khan M. (2012). Effects of fruits extracts of three medicinal plants on the blood glucose level of glucose-induced hyperglycemic and normal rabbit models. *Journal of Biology and Life Science*.

[7] Rader J. I., Calvert R. J., Hathcock J. N. (1992). Hepatic toxicity of unmodified and time-release preparations of niacin. *The American Journal of Medicine*.

[8] Sabir M., Akhter M., Bhide N. (1978). Further studies on pharmacology of berberine. *Indian Journal of Physiology and Pharmacology*.

[9] Belwal T., Bisht A., Devkota H. P. (2020). Phytopharmacology and clinical updates of Berberis species against diabetes and other metabolic diseases. *Frontiers in Pharmacology*.

[10] Ivanovska N., Philipov S. (1996). Study on the anti-inflammatory action of *Berberis vulgaris* root extract, alkaloid fractions and pure alkaloids. *International Journal of Immunopharmacology*.

[11] Fatehi M., Saleh T. M., Fatehi-Hassanabad Z., Farrokhfal K., Jafarzadeh M., Davodi S. (2005). A pharmacological study on *Berberis vulgaris* fruit extract. *Journal of Ethnopharmacology*.

[12] Yin J., Hu R., Chen M. (2002). Effects of berberine on glucose metabolism in vitro. *Metabolism*.

[13] Doggrell S. A. (2005). Berberine-a novel approach to cholesterol lowering. *Expert Opinion on Investigational Drugs*.

[14] Kong W., Wei J., Abidi P. (2004). Berberine is a novel cholesterol-lowering drug working through a unique mechanism distinct from statins. *Nature Medicine*.

[15] Ashraf H., Heidari R., Nejati V. (2014). Antihyperglycemic and antihyperlipidemic effects of fruit aqueous extract of *Berberis integerrima* Bge. in streptozotocin-induced diabetic rats. *Iranian Journal of Pharmaceutical Research*.

[16] Imenshahidi M., Hosseinzadeh H. (2016). *Berberis vulgaris* and berberine: an update review. *Phytotherapy Research*.

[17] Yin J., Xing H., Ye J. (2008). Efficacy of berberine in patients with type 2 diabetes mellitus. *Metabolism*.

[18] Sabahi Z., Khoshnood-Mansoorkhani M. J., Namadi S. R., Moein M. (2016). Antidiabetic and synergistic effects study of anthocyanin fraction from *Berberis integerrima* fruit on streptozotocin-induced diabetic rats model. *Trends in Pharmacological Sciences*.

[19] Fallah H., Akbari H., Abolhassani M., Mohammadi A., Gholamhosseinian A. (2017). *Berberis integerrima* ameliorates insulin resistance in high-fructose-fed insulin-resistant rats. *Iranian Journal of Basic Medical Sciences*.

[20] Vafadar F., Amooaghaie R., Ehsanzadeh P., Ghanadian M., Talebi M., Ghanati F. (2020). Melatonin and calcium modulate the production of rosmarinic acid, luteolin, and apigenin in *Dracocephalum kotschyi* under salinity stress. *Phytochemistry*.

[21] Soltani R., Gorji A., Asgary S., Sarrafzadegan N., Siavash M. (2015). Evaluation of the effects of *Cornus mas* L. fruit extract on glycemic control and insulin level in type 2 diabetic adult patients: a randomized double-blind placebo-controlled clinical trial. *Evidence-Based Complementary and Alternative Medicine*.

[22] Sanjari M., Shamsinejad B., Khazaeli P., Safi Z., Mirrashidi F., Naghibzadeh-Tahami A. (2020). Safety and efficacy of *Berberis integerrima* root extract in patients with type 2 diabetes. A parallel intervention based triple blind clinical trial. *Journal of Diabetes & Metabolic Disorders*.

[23] Aryaeian N., Khorshidi Sedehi S., Khorshidi M., Zarezadeh M., Hosseini A. F., Shahram F. (2020). Effects of hydroalcoholic extract of *Berberis integerrima* on the anthropometric indices and metabolic profile in active rheumatoid arthritis patients. *Complementary Therapies in Medicine*.

[24] Bayani M., Ahmadi-hamedani M., Jebelli Javan A. (2016). Phytochemical and antioxidant activities of *Berberis integerrima* and *Berberis vulgaris* and pharmacological effects of the more active species on alloxan-induced diabetic rats. *Journal of Medicinal Plants*.

[25] Shidfar F., Ebrahimi S. S., Hosseini S., Heydari I., Shidfar S., Hajhassani G. (2012). The effects of *Berberis vulgaris* fruit extract on serum lipoproteins, apob, apoa-i, homocysteine, glycemic control and total antioxidant capacity in type 2 diabetic patients. *Iranian Journal of Pharmaceutical Research*.

[26] Hemmati M., Serki E., Gholami M., Hoshyar R. (2017). Effects of an ethanolic extract of *Berberis vulgaris* fruits on hyperglycemia and related gene expression in streptozotocin-induced diabetic rats. *Clinical Phytoscience*.

[27] Maritim A. C., Sanders R. A., Watkins J. B. (2003). Diabetes, oxidative stress, and antioxidants: a review. *Journal of Biochemical and Molecular Toxicology*.

[28] Siow Y. L., Sarna L., Karmin O. (2011). Redox regulation in health and disease—therapeutic potential of berberine. *Food Research International*.

[29] Yang Y., Ling W., Yang Y. (2017). Role of purified anthocyanins in improving cardiometabolic risk factors in Chinese men and women with prediabetes or early untreated diabetes-a randomized controlled trial. *Nutrients*.

[30] Meliani N., Dib M. E. A., Allali H., Tabti B. (2011). Hypoglycaemic effect of *Berberis vulgaris* L. in normal and streptozotocin-induced diabetic rats. *Asian Pacific Journal of Tropical Biomedicine*.

[31] Afsharinasab M., Mohammad-Sadeghipour M., Hajizadeh M. R., Khoshdel A., Mirzaiey V., Mahmoodi M. (2020). The effect of hydroalcoholic *Berberis integerrima* fruits extract on the lipid profile, antioxidant parameters and liver and kidney function tests in patients with nonalcoholic fatty liver disease. *Saudi Journal of Biological Sciences*.

[32] Hwang K. H., Ahn J. Y., Kim S., Ha T. Y. (2009). Anti-obesity effects of berberine in mice fed a high fat diet. *Journal of Food Science & Nutrition*.

